# Dosage Frequency Effects on Treatment Outcomes Following Self-managed Digital Therapy: Retrospective Cohort Study

**DOI:** 10.2196/36135

**Published:** 2022-07-20

**Authors:** Claire Cordella, Michael Munsell, Jason Godlove, Veera Anantha, Mahendra Advani, Swathi Kiran

**Affiliations:** 1 Department of Speech Language and Hearing Sciences Boston University Boston, MA United States; 2 Constant Therapy Health Lexington, MA United States

**Keywords:** aphasia, stroke, technology, rehabilitation, dosage

## Abstract

**Background:**

Although the efficacy of high-dose speech-language therapy (SLT) for individuals with poststroke aphasia has been established in the literature, there is a gap in translating these research findings to clinical practice. Therefore, patients continue to receive suboptimal amounts of SLT, with negative consequences for their functional communication recovery. Recent research has identified self-managed digital health technology as one way to close the dosage gap by enabling high-intensity therapy unrestricted by clinician availability or other practical constraints. However, there is limited empirical evidence available to rehabilitation professionals to guide dose prescriptions for self-managed SLT despite their increasing use in the COVID-19 era and likely beyond.

**Objective:**

This study aims to leverage real-world mobile health data to investigate the effects of varied dosage frequency on performance outcomes for individuals with poststroke speech, language, and cognitive deficits following a 10-week period of self-managed treatment via a commercially available digital health platform.

**Methods:**

Anonymized data from 2249 poststroke survivors who used the Constant Therapy app between late 2016 and 2019 were analyzed. The data included therapy tasks spanning 13 different language and cognitive skill domains. For each patient, the weekly therapy dosage was calculated based on the median number of days per week of app use over the 10-week therapy period, binned into groups of 1, 2, 3, 4, or ≥5 days per week. Linear mixed-effects models were run to examine change in performance over time as a function of dosage group, with post hoc comparisons of slopes to evaluate the performance gain associated with each additional day of practice.

**Results:**

Across all skill domains, linear mixed-effects model results showed that performance improvement was significantly greater for patients who practiced 2 (*β*=.001; *t*_15,355_=2.37; *P*=.02), 3 (*β*=.003; *t*_9738_=5.21; *P*<.001), 4 (*β*=.005; *t*_9289_=7.82; *P*<.001), or ≥5 (*β*=.005; *t*_6343_=8.14; *P*<.001) days per week compared with those who only practiced for 1 day per week. Post hoc comparisons confirmed an incremental dosage effect accumulating with each day of practice (ie, 1 day vs 2 days, 2 days vs 3 days, and 3 days vs 4 days), apart from 4 days versus ≥5 days of practice per week. The result of greater improvement for higher versus lower dosage frequency groups was true not only across all domains but also within a majority of individual subdomains.

**Conclusions:**

The findings from this study demonstrated that increased dosage frequency is associated with greater therapy gains over a 10-week treatment period of self-managed digital therapy. The use of real-world data maximizes the ecological validity of study results and makes the findings more generalizable to clinical settings. This study represents an important step toward the development of optimal dose recommendations for self-managed SLT.

## Introduction

### Background

Approximately one-third of all strokes result in aphasia or other communication disorders that affect a person’s ability to speak, understand, read or write [1]. For a significant number of stroke survivors with aphasia—an estimated 2.25 million in the United States and the United Kingdom [1]—these communication deficits portend poorer global health outcomes (compared with stroke survivors without aphasia), including higher overall mortality, reduced functional recovery, social isolation, and reduced overall quality of life [2-5].

Fortunately, speech-language therapy (SLT) is an effective means for improving impairment- and participation-based language outcomes in individuals with chronic poststroke aphasia. A comprehensive Cochrane review of SLT randomized control trials reported greater benefits to communication when patients with chronic aphasia received therapy at high intensity (from 4 to 15 hours per week), high dosage (27-208 hours in total), or over a long period (up to 22 months) compared to more moderate treatment schedules [6].

Despite the evidence that supports the provision of high-dose SLT to stroke survivors with aphasia, patients are often unable to access sufficiently intense therapy as part of their usual care. Across the English-speaking world, it is estimated that individuals with chronic poststroke aphasia receive, on average, <5 hours of therapy per week [7,8], far less than the recommended 5 to 10 hours per week that is typical of evidence-based intensive therapy regimens [6,9]. This reality of insufficient usual care is caused by several barriers that practically limit patients’ access to SLT, such as provider shortages, caps on Medicare reimbursement, geographic isolation, and lack of transportation, among other factors [10,11].

One way to offset the lack of sufficient therapy is to enable patients to engage in in-home practice through computerized or app-based therapeutic programs [11,12]. Many studies have evaluated digital SLT interventions as part of a treatment protocol, delivered as tablet- or computer-based programs [13-26]. A smaller subset of studies have investigated self-managed programs, in which users not only complete therapy at home but also determine their own practice schedule [22-26]. Crucially, the freedom to determine one’s own practice schedule means that dose parameters for these types of therapies can and do vary widely from patient to patient [27]. This naturally occurring variance in dosage presents a unique opportunity to probe dose-response relationships in SLT. Dose articulation studies are a critical first step toward establishing optimal dosage recommendations for SLT interventions [28,29]. To date, only a handful of studies have directly compared different dosage amounts of the same intervention, and none have done so in the context of self-managed digital therapies [30-36].

### Objective

In this study, we leveraged real-world mobile health data to investigate the effects of different dose levels on treatment outcomes following a 10-week treatment period using a commercially available digital health platform. Noting that a consensus on the definition of SLT dosage and intensity has not been definitively reached in the literature [28,29,37-39], we chose to focus on dosage frequency, which is defined as the number of days per week during which a patient completes a therapy session. This measure is easily generalizable across patients and applicable to clinical settings. In this analysis, we retrospectively examined how often users completed computer-based therapy sessions with the Constant Therapy program and evaluated the relationship between their dosage frequency and improvement over the treatment period in several functional domains. It was hypothesized that patients who adhered to a greater dosage frequency of therapy would see greater improvement during the first 10 weeks of treatment than individuals at the lowest dosage frequency.

## Methods

### Participants

Data were aggregated and analyzed from patients who used the Constant Therapy app between October 2016 and October 2019. The data were anonymized before being shared for analysis with Boston University. All users (N=238,767) consented to the use of their exercise and therapy performance data for research purposes. Constant Therapy users were asked to provide basic demographic and diagnostic information upon initial sign-up, including age, time since injury, sex, and diagnoses (eg, stroke, aphasia, and traumatic brain injury). For this study, only users who reported having had a stroke with resultant speech, language, and cognitive deficits were included for analysis. An additional inclusion criterion was applied that required users to engage with the app for at least one day in 10 of their first 15 calendar weeks of use. The resultant study sample included 2249 unique patients with speech, language, and cognitive deficits following stroke. Across the entire sample and within each dosage group, the most commonly endorsed diagnoses were stroke alone; stroke and aphasia; and stroke, aphasia, and apraxia. Dosage groups were determined by first calculating, per individual, the median number of days per week of Constant Therapy use over the 10-week therapy period of interest, and then binning into categories of 1, 2, 3, 4, or ≥5 days per week.

### Therapy Program

Constant Therapy [40] is an evidence-based digital therapeutic that features over 244 individual tasks spanning various speech, language, and cognitive skill domains [22,25,26,41,42]. This study focused on task data for the following 13 domains: (1) auditory comprehension, (2) phonological processing, (3) production, (4) reading, (5) writing, (6) naming, (7) attention, (8) auditory memory, (9) visual memory, (10) analytical, (11) arithmetic, (12) quantitative, and (13) visuospatial skills. Importantly, users can tailor their therapy program by self-selecting the skill domains in need of improvement, meaning that the specific tasks being worked on as part of therapy differ from user to user. Task difficulty is also adjusted on a user-by-user basis, based on an adaptive difficulty algorithm that advances users to a more difficult version of a given task once they have achieved mastery. During a session, patients practice tasks in order of increasing level of difficulty. The order in which subsequent, more difficult tasks are assigned is determined by a universal task progression order per domain, whereby every task type is ranked serially from least to most difficult. The progression order for each skill domain was structured based on research evidence and clinician consultation and fine-tuned using population performance [43].

**Figure 1:**
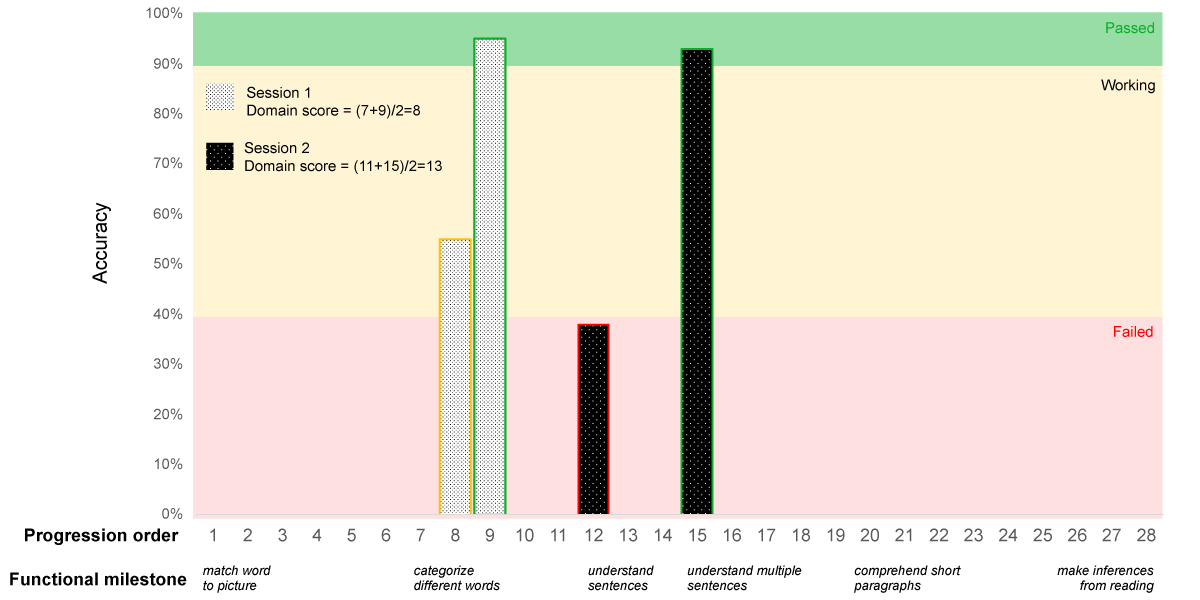
Example calculated domain score (reading domain). Tasks are introduced to a patient according to a domain’s progression order (x-axis, top row). The level of function a patient should be able to demonstrate after successfully passing the listed task is noted as the functional milestone (x-axis, bottom row). Shaded bars show the task accuracy scores for a hypothetical patient’s highest task passed and lowest task working or failed across 2 different sessions.

Within each practice session, each task is practiced until accuracy reaches 90% or higher on ≥2 occasions, at which point a patient is advanced to the next level of difficulty or to a different task. In addition, if a user was not improving on a level or their accuracy was below 40%, a lower level of the task was assigned in addition to or in replacement of the original task. The Constant Therapy program records task performance data (ie, accuracy) and session activities, including usability logs, time stamps, and item completion indicators. On the basis of these performance data, a domain score is calculated to provide a summative assessment of a user’s performance in a specific skill domain, considering that users are completing tasks at various difficulty levels. Generation of the domain score involves (1) identifying the highest task passed (accuracy ≥90%) or working (accuracy between 40% and 90%) and the lowest task working or failed (accuracy <40%) during a session and (2) taking the average progression order of the highest level passed or working and the lowest level working or failed, thereby providing an estimate of the given session’s difficulty level. The progression order for failed or working tasks is adjusted by subtracting 1, because the highest difficulty level successfully passed at that time is represented by the previous task in the progression order. Scores are normalized by dividing by the total number of task levels—which varies by domain—to make scores comparable across different skill domains. Therefore, change in domain score can be interpreted as a patient’s increase in difficulty level as a percentage of a domain’s total items. An example domain score calculation for a hypothetical patient in the reading domain is provided in Figure 1. Domain scores were averaged across sessions if multiple sessions occurred in a single week.

This study used the calculated weekly domain scores for 2 purposes. First, weekly domain scores served as the dependent variable of interest to index performance change over the intervention period. The weekly domain score was the average domain score across all sessions completed by the user per week, calculated for each of the 10 weeks of the intervention period. Second, we extracted the domain score at baseline (ie, week 0 of the intervention period) as a means by which to index initial severity, as standardized measures of baseline language and cognitive function were not available in the data set, given the real-world nature of the data obtained.

### Statistical Analysis

For each of the 13 domains, the first week of therapy was treated as the baseline (assigned as week 0), and weekly domain scores were extracted for each of the 10 weeks of the intervention period, as described earlier. In order to examine changes in weekly domain scores over time as a function of dosage frequency group, linear mixed-effects models (LMMs) were run first for scores combined across all domains and then independently for each domain. For the overall model encompassing all domains, the weekly domain score served as the dependent variable, with fixed effects of time (week number), dosage frequency group, cumulative practice amount (ie, total hours spent completing therapy tasks), time × dosage frequency group, and time × cumulative practice amount. Covariates of age, time since stroke (≤6 and >6 months), sex, and baseline domain scores were also included as fixed effects in the model. The model included random effects of patients and domains. This final model structure was determined through an iterative process of stepwise addition of model terms, beginning with the determination of the optimal random effects structure and proceeding to the determination of the optimal fixed-effects structure. Nested models were compared using Likelihood-Ratio Tests with additional reference to the Akaike information criterion and Bayesian information criterion values of each candidate model. The LMM building and selection process is reported in detail in Multimedia Appendix 1, following best-practice conventions for LMM reporting in psychological science [44]. This same model structure was applied to the analyses of each of the 13 individual domains, except that for these analyses, the random effect of the domain was excluded. All statistical analyses were conducted in R (version 4.0.2; R Foundation for Statistical Computing) using *lme4*, *lmerTest*, *emmeans*, and *sjPlot* packages [45-49].

### Ethics Approval

This project was considered an institutional review board–exempt retrospective analysis by Pearl Institutional Review Board (#17-LNCO-101) under 45 Code of Federal Regulations 46.101(b) category 2.

## Results

### All Skill Domains

Data of 2249 patients with poststroke deficits in speech, language, or cognitive deficits were analyzed in this study. The average age of the sample was 63 (SD 14) years, and the majority of patients (N=1319) were in the acute recovery stage (ie, ≤6 months poststroke). The average (normalized) baseline domain score was 33% (SD 20%), indicating that the patients were typically in the lower third of the domain’s task progression order during their first week of therapy. The dosage groups did not significantly differ in terms of age, sex, or proportion of patients with acute condition. With regard to age, digital literacy did not appear to be a barrier to use among older adults in the sample, as older users showed similar practice patterns as younger users, in line with previously published findings showing robust engagement with the Constant Therapy app among older users [27]. Significant overall differences across dosage frequency groups were observed for baseline domain score (*F*_4,57898_=6.937; *P*<.001) and total hours of therapy (*F*_4,61197_=54.54; *P*<.001), although effect sizes between dosage groups were uniformly small for both measures (Multimedia Appendix 1). Nonetheless, these factors were included as covariates in all analysis models to account for the potential confounding effects of severity (ie, baseline domain score) and cumulative therapy exposure (ie, total hours of therapy). The summary statistics for the entire cohort and for each dosage frequency group are presented in Table 1.

**Table 1:** Summary statistics of study cohort (N=2249).

Characteristics		Overall (N=2249)			By dosage frequency group								
					1 day per week (N=888)		2 days per week (N=1155)		3 days per week (N=804)		4 days per week (N=574)		5 days per week (N=481)
Age (years), mean (SD)			63 (14)	64 (14)		64 (14)		63 (13)		63 (13)		63 (13)	
**Sex, n (%)**													
	Male		1269 (56.4)	500 (56.3)		645 (55.8)		459 (57.1)		335 (58.4)		277 (57.6)	
	Female		968 (43)	384 (43.2)		506 (43.8)		343 (42.7)		236 (41.1)		199 (41.4)	
	Not specified		12 (0.5)	4 (0.5)		4 (0.3)		2 (0.2)		3 (0.5)		5 (1)	
**Chronicity, n (%)**													
	Acute (≤6 months)		1319 (58.6)	494 (55.6)		671 (58.1)		463 (57.6)		335 (58.4)		294 (61.1)	
	Chronic (>6 months)		930 (41.4)	394 (44.4)		484 (41.9)		341 (42.4)		239 (41.6)		187 (38.9)	
Baseline domain score^a^, mean (SD)			0.33 (0.20)	0.33 (0.21)		0.33 (0.20)		0.34 (0.20)		0.34 (0.20)		0.34 (0.20)	
Total hours^a^, mean (SD)			6.2 (22)	3.7 (21.4)		5.3 (28.6)		6.1 (22.8)		6.9 (11.4)		10.6 (11.9)	

^a^Baseline domain score and total hours variables are calculated per individual skill domain.

Across all skill domains, the model results (Tables 2 and 3) revealed significant main effects of time (*F*_1,15_=106.46; *P*<.001), time since stroke (*F*_1,1753_=16.57; *P*<.001), baseline domain score (*F*_1,104,365_=67,301.21; *P*<.001), cumulative practice amount (*F*_1,60502_=12.83; *P*<.001), and dosage group frequency (*F*_4,8873_=6.22; *P*<.001) on domain score in the 10-week treatment period. Specifically, a greater weekly domain score was associated with an increase in the number of weeks of therapy (*β*=.009; *t*=−12.27; *P*<.001), acute condition (*β*=.010; *t*=4.07; *P*<.001), higher baseline domain score (*β*=.662; *t*=259.42; *P*<.001), greater cumulative practice amount (*β*=.0001; *t*=3.58; *P*<.001), and greater practice frequency (2 days: *β*=.001, *t*=0.52, *P*=.60; 3 days: *β*=.008, *t*=3.16, *P*=.002; 4 days: *β*=.008, *t*=2.91, *P*=.004; ≥5 days: *β*=.011, *t*=3.95, *P*<.001). Age and sex were not significant predictors of domain score, nor was the interaction of time × cumulative practice amount.

**Figure 2:**
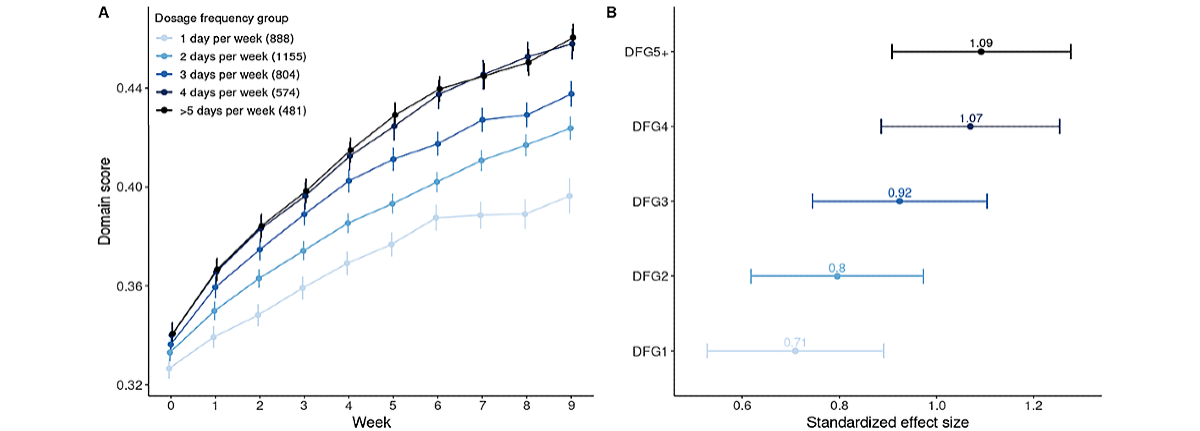
Change in domain score as a function of dosage frequency group, across all skill domains. (A) The average weekly domain score improved over the treatment period for all dosage frequency groups, but the rate of improvement was significantly greater for the higher versus lower dosage groups. Numbers in parentheses in the legend correspond to the number of unique patients in each dosage frequency group. Error bars represent the SE of the mean. (B) The treatment effect sizes were greater for the higher versus lower dosage groups. DFG: dosage frequency group (1 day per week, 2 days per week, 3 days per week, 4 days per week, ≥5 days per week).

Crucial to our question of interest, the time × dosage frequency group interaction was significant (*F*_4,10347_=6.22; *P*<.001), indicating that although we see gains in domain score for all dosage groups over time (Figure 2A), the rate of improvement is highly dependent on the frequency of practice. Rates of improvement were significantly greater for patients who practiced 2 (*β*=.001; *t*_15,355_ =2.37; *P*=.02), 3 (*β*=.003; *t*_9738_=5.21; *P*<.001), 4 (*β*=.005; *t*_9289_=7.82; *P*<.001), or ≥5 (*β*=.005; *t*_6343_=8.14; *P*<.001) days per week than for those who only practiced 1 day per week. Furthermore, post hoc pairwise comparison of slopes (Table 4) showed an incremental dosage effect accumulating with each additional day of practice (ie, 1 day vs 2 days, 2 days vs 3 days, and 3 days vs 4 days), apart from 4 days versus ≥5 days a week of practice. Table 5 presents a pairwise comparison of estimated means per dosage frequency group at the beginning (ie, week 0) and end (ie, week 9) of treatment, illustrating that although at baseline, domain scores between incremental dosage groups (ie, 1 day vs 2 days, 2 days vs 3 days, 3 days vs 4 days, and 4 days vs ≥5 days) were not significant, by the end of treatment, significant differences in means emerged for all group comparisons except for the groups practicing 4 days versus ≥5 days per week. This result indicates that the significant magnitude differences accrued over the course of treatment are attributable to differences in slopes across the dosage groups as opposed to baseline differences in means. Figure 2B shows the cumulative effect of treatment—calculated as the standardized pretreatment versus posttreatment effect size per dosage group—and demonstrates that although there was at least a moderate treatment effect for all dosage groups, this effect was larger for patients who practiced more frequently. The standardized effect size was calculated for each dosage frequency group based on the difference in LMM-generated estimated marginal means from pretreatment (ie, week 0) to posttreatment (ie, week 9), using the eff_size function in the *emmeans* package in R.

**Table 2:** Final linear mixed-effects model results summary (fixed effects), across all skill domains^a,b^.

Predictors			Estimates (SE)		*t* test (*df*)		*P* value
**Fixed effects**							
	Intercept	1.36×10^−1^ (1.18×10^−2^)		12.27 (2.51×10^1^)		*<.001^c^*	
	Week	9.28×10^−3^ (1.22×10^−3^)		7.62 (1.73×10^1^)		*<.001*	
	Dosage group (2 days per week)	1.15×10^−3^ (2.22×10^−3^)		0.52 (1.34×10^4^)		.60	
	Dosage group (3 days per week)	8.00×10^−3^ (2.54×10^−3^)		3.16 (8.21×10^3^)		.002	
	Dosage group (4 days per week)	8.30×10^−3^ (2.85×10^−3^)		2.91 (7.91×10^3^)		.004	
	Dosage group (≥5 days per week)	1.13×10^−2^ (2.86×10^−3^)		3.95 (5.09×10^3^)		*<.001*	
	Total hours	1.13×10^−4^ (3.15×10^−5^)		3.58 (6.05×10^4^)		*<.001*	
	Domain score baseline	6.62×10^−1^ (2.55×10^−3^)		259.42 (1.04×10^5^)		*<.001*	
	Age (years)	−1.54×10^−4^ (8.96×10^−5^)		−1.72 (1.79×10^3^)		.09	
	Sex (male)	−1.39×10^−4^ (2.43×10^−3^)		−0.06 (1.78×10^3^)		.95	
	Sex (not specified)	2.13×10^−2^ (1.66×10^−2^)		1.28 (1.83×10^3^)		.20	
	Chronicity (acute)	9.93×10^−3^ (2.44×10^−3^)		−4.07 (1.75×10^3^)		*<.001*	
	Week × dosage group (2 days per week)	1.13×10^−3^ (4.78×10^−4^)		2.37 (1.54×10^4^)		.02	
	Week × dosage group (3 days per week)	2.82×10^−3^ (5.42×10^−4^)		5.21 (9.74×10^3^)		*<.001*	
	Week × dosage group (4 days per week)	4.73×10^−3^ (6.05×10^−4^)		7.82 (9.29×10^3^)		*<.001*	
	Week × dosage group (≥5 days per week)	5.03×10^−3^ (6.17×10^−4^)		8.14 (6.34×10^3^)		*<.001*	
	Week × total hours	6.01×10^−6^ (4.78×10^−6^)		0.97 (6.50×10^4^)		.33	

^a^N (total observations)=111,768; N (patients)=2249; N (domains)=13.

^b^Model equation: domain score (weekly average) ~ week × (dosage group + total hours) + baseline domain score + age + sex + chronicity + (1+ week:patient) + (1+ week:domain).

^c^Italicized text indicates a significant predictor, *P*<.001.

**Table 3:** Final linear mixed-effects model results summary (random effects), across all skill domains^a,b^.

Predictors			Variance (SD)		Correlation
**Random effects**					
	Residual	1.4×10^−2^ (1.2×10^−1^)		N/A^c^	
	Patient (intercept)	2.2×10^−3^ (4.7×10^−2^)		N/A	
	Domain (intercept)	1.3×10^−3^ (3.6×10^−2^)		N/A	
	Week:patient (slope)	1.5×10^−5^ (1.2×10^−2^)		5.2×10^−1^	
	Week:domain (slope)	1.6×10^−5^ (4.0×10^−3^)		3.0×10^−2^	

^a^N (total observations)=111,768; N (patients)=2249; N (domains)=13.

^b^Model equation: domain score (weekly average) ~ week × (dosage group + total hours) + baseline domain score + age + sex + chronicity + (1+ week:patient) + (1+ week:domain).

^c^N/A: not applicable.

**Table 4:** Pairwise comparisons of slopes by dosage frequency group^a^.

Contrast			Estimate (SE)		*t* test (*df*^b^)		*P* value^c^
**1 day per week**							
	2 days per week	−1.13×10^−3^ (4.77×10^−4^)		−2.37 (infinity)		.12	
	3 days per week	−2.82×10^−3^ (5.42×10^−4^)		−5.21 (infinity)		*<.001* ^d^	
	4 days per week	−4.73×10^−3^ (6.05×10^−4^)		−7.82 (infinity)		*<.001*	
	5 days per week	−5.03×10^−3^ (6.17×10^−4^)		−8.14 (infinity)		*<.001*	
**2 days per week**							
	3 days per week	−1.69×10^−3^ (4.46×10^−4^)		−3.79 (infinity)		*.001*	
	4 days per week	−3.60×10^−3^ (5.30×10^−4^)		−6.79 (infinity)		*<.001*	
	5 days per week	−3.90×10^−3^ (5.49×10^−4^)		−7.10 (infinity)		*<.001*	
**3 days per week**							
	4 days per week	−1.91×10^−3^ (5.16×10^−4^)		−3.70 (infinity)		*.002*	
	5 days per week	−2.20×10^−3^ (5.56×10^−4^)		−3.97 (infinity)		*.001*	
**4 days per week**							
	5 days per week	−2.94×10^−4^ (5.56×10^−4^)		−0.52 (infinity)		.99	

^a^Results are averaged over the levels of sex and chronicity.

^b^Degrees-of-freedom method: asymptotic.

^c^*P* value adjustment: Tukey method for comparing a family of 5 estimates.

^d^Italicized text indicates significant contrast, *P*<.05.

**Table 5:** Pairwise comparison of estimated marginal means at beginning and end of treatment.

Contrast			Estimate (SE)	*t* test (*df*)	*P* value
**Week 0 (baseline)**					
	**1 day per week**				
		2 days per week	−1.15×10^−3^ (2.22×10^−3^)	−0.52 (infinity)	.99
		3 days per week	−8.00×10^−3^ (2.53×10^−3^)	−3.16 (infinity)	*.01^a^*
		4 days per week	−8.30×10^−3^ (2.85×10^−3^)	−2.91 (infinity)	*.03*
		5 days per week	−1.13×10^−2^ (2.86×10^−3^)	−3.95 (infinity)	*.001*
	**2 days per week**				
		3 days per week	−6.85×10^−3^ (2.20×10^−3^)	−3.12 (infinity)	*.02*
		4 days per week	−7.15×10^−3^ (2.60×10^−3^)	−2.75 (infinity)	*.047*
		5 days per week	−1.01×10^−2^ (2.63×10^−3^)	−3.86 (infinity)	*.001*
	**3 days per week**				
		4 days per week	−2.95×10^−4^ (2.61×10^−3^)	−0.11 (infinity)	.99
		5 days per week	−3.30×10^−3^ (2.73×10^−3^)	−1.21 (infinity)	.75
	**4 days per week**				
		5 days per week	−3.00×10^−3^ (2.85×10^−3^)	−1.05 (infinity)	.83
**Week 9 (end of analysis period)**					
	**1 day per week**				
		2 days per week	−1.13×10^−2^ (3.18×10^−3^)	−3.57 (infinity)	*.003*
		3 days per week	−3.34×10^−2^ (3.78×10^−3^)	−8.84 (infinity)	*<.001*
		4 days per week	−5.09×10^−2^ (4.27×10^−3^)	−11.92 (infinity)	*<.001*
		5 days per week	−5.65×10^−2^ (4.63×10^−3^)	−12.20 (infinity)	*<.001*
	**2 days per week**				
		3 days per week	−2.21×10^−2^ (2.85×10^−3^)	−7.74 (infinity)	*<.001*
		4 days per week	−3.95×10^−2^ (3.54×10^−3^)	−11.16 (infinity)	*<.001*
		5 days per week	−4.52×10^−2^ (4.00×10^−3^)	−11.28 (infinity)	*<.001*
	**3 days per week**				
		4 days per week	−1.75×10^−2^ (3.18×10^−3^)	−5.49 (infinity)	*<.001*
		5 days per week	−2.31×10^−2^ (3.84×10^−3^)	−6.02 (infinity)	*<.001*
	**4 days per week**				
		5 days per week	−5.65×10^−3^ (3.67×10^−3^)	−1.54 (infinity)	.54

^a^Italicized text indicates significant contrast, *P*<.05.

### Individual Skill Domains

**Figure 3:**
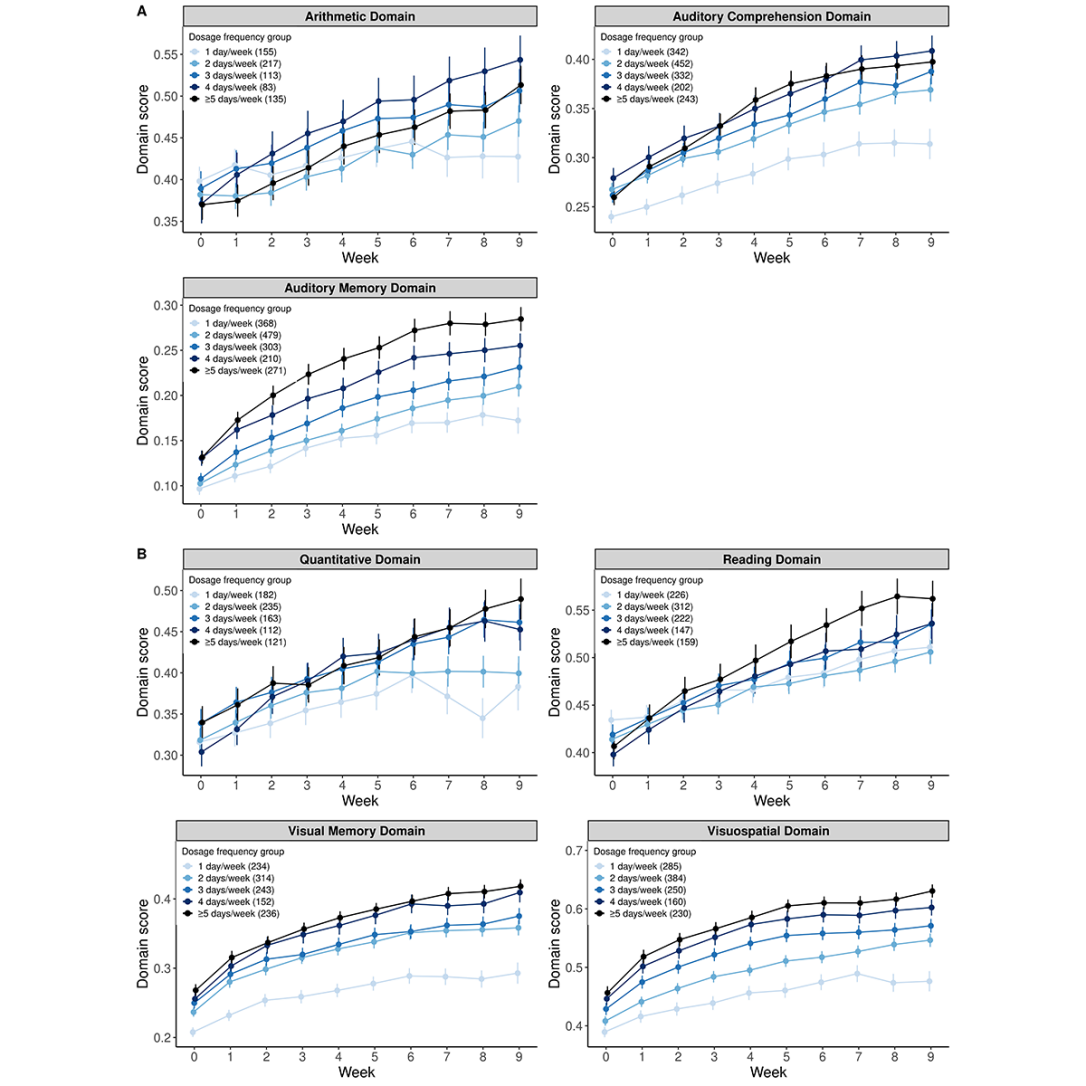
Weekly change in domain score as a function of dosage frequency group, by skill domain (significant time × dosage group effect). (A) Arithmetic, auditory comprehension, and auditory memory domains showed a significantly greater rate of change in domain scores for practice frequencies of 2, 3, 4, and ≥5 days per week compared with 1 day per week. (B) Quantitative, reading, visual memory, and visuospatial domains showed a significantly greater rate of change in domain scores for practice frequencies of 4 and ≥5 days per week. compared with 1 day per week.

**Figure 4:**
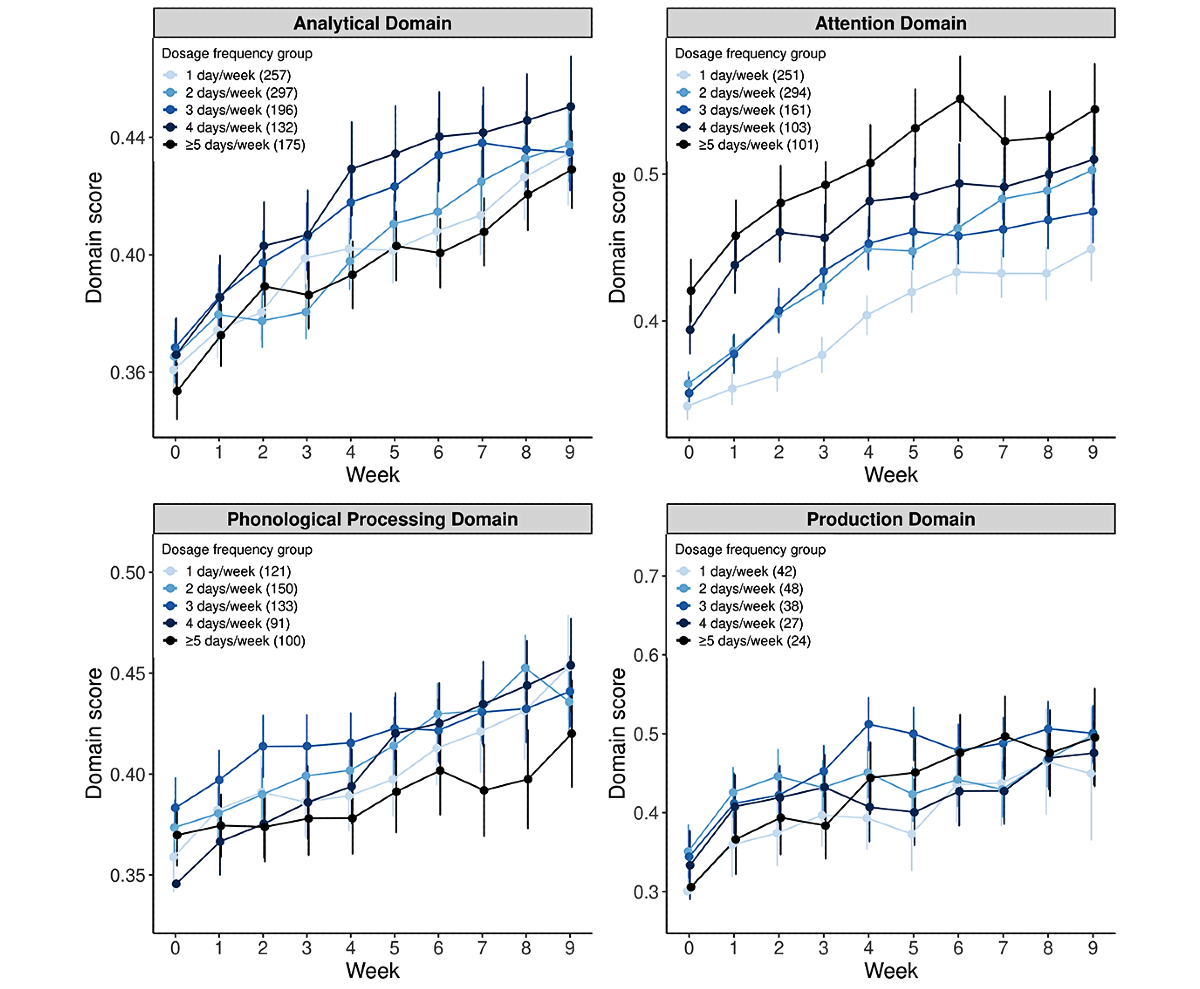
Weekly change in domain score as a function of dosage frequency group, by skill domain (nonsignificant time×dosage group effect).

Within individual skill domains, the hypothesis that patients with a greater dosage frequency see greater improvement over time is supported by the majority of individual domain models. Specifically, separate LMMs similarly revealed a significant time × dosage frequency group interaction for 9 of the 13 total domains. These 9 domains included the arithmetic, auditory comprehension, auditory memory, naming, quantitative, reading, visual memory, visuospatial, and writing domains (Figure 3; Multimedia Appendix 1). For most of these domains, model results revealed a trend similar to the overall model results, in which a significantly greater rate of change in domain score was observed for higher versus lower practice frequencies (Multimedia Appendix 1). For the arithmetic, auditory comprehension, and auditory memory domains, there was a significantly greater rate of change in domain score for practice frequencies of 2, 3, 4, and ≥5 days per week than that of 1 day per week (Figure 3A). For the quantitative, reading, visual memory, and visuospatial domains, there was a significantly greater rate of change in the domain score for practice frequencies of 4 and ≥5 days per week than that of 1 day per week (Figure 3B). In the naming and writing domains, despite an overall significant interaction between time and dosage frequency group, the slope was statistically significant only for 4 days per week and 3 days per week (compared with 1 day per week) dosage frequency groups (Multimedia Appendix 1). For the remaining domains—analytical, attention, phonological processing, and production—no significant interaction between time and dosage frequency group was observed, indicating that improvement over the treatment period did not differ based on practice frequency (Figure 4; Multimedia Appendix 1).

## Discussion

### Summary of Findings

Patients with poststroke speech, language, and cognitive impairment generally saw an improvement in their ability to accurately perform tasks of increasing difficulty during their first 10 weeks of self-managed digital therapy across a variety of skill domains. Overall, for most of the individual domains assessed, the rate of improvement was modulated by practice frequency, with significantly greater improvement over time for patients who practiced 3 to 5 days per week than patients who only completed sessions 1 day per week.

These results are generally concordant with the growing body of literature showing the benefits of high-dose and high-intensity SLT on communication outcomes [6,50]. Our work adds to this research by providing results for a large sample over a broad range of functional skill domains. It also underscores the importance of delineating the component parameters of dosage. Increasingly, in the literature, cumulative intervention intensity (ie, total dose) is quantified as the product of session dose, frequency, and intervention duration [29,37,38,51]. In this study, our primary research questions were focused on dosage frequency; but we also accounted for dose amount and duration. Importantly, the results for dosage frequency presented here are independent of overall duration and total number of hours of therapy and therefore underscore the importance of considering practice frequency in addition to other related dose parameters when devising optimal dosage recommendations. Furthermore, the model results showed that although the main effect of the total amount of therapy was significant, it did not predict the rate of improvement over time in the same way that dosage frequency did. This finding demonstrates that, although different dose parameters may be related, they do not necessarily have an equal impact on treatment outcomes.

In this study, analyses were conducted on real-world patient usage data that included a wide range of dosage frequencies, from 1 day per week up to ≥5 days per week. This ensured that the dosage frequencies being investigated are practically achievable. Recent work has identified a major gap between the dose parameters being studied in research—which tend to be uniformly high—and the modest therapy doses being delivered as part of routine clinical practice [51,52]. For example, a study of dosage amounts in a US-based outpatient setting reported a median total therapy dosage of just 7.5 hours over a median 7.7-week treatment duration for individuals with poststroke aphasia, compared with a significantly more intensive dosage regimen (median 20 hours over a median 6-week period) reported in the aphasia treatment literature from 2009 to 2019 [51]. This dosage gap is a major barrier to the successful clinical implementation of research findings. Thus, investigating naturally occurring dosage frequencies maximizes ecological validity, and by extension, the potential for findings to directly inform clinical dose recommendations. Analysis of a range of dosage frequencies is also important because it allowed for post hoc comparison of individual dosage frequency groups (1 day vs 2 days per week, 2 days vs 3 days per week, etc). The results demonstrated that, across all domains, each additional day of practice per week was associated with a significantly greater improvement over time, with the exception of 4 versus ≥5 days per week. The nonsignificant difference in performance outcome at the upper end of the practice frequency range raises the possibility of diminishing returns, a finding that has also been suggested in other recent work and may be explained by a ceiling effect for certain impairment-based therapies [30]. The existence of a lower threshold for improvement is similarly a source of debate in the limited available literature; for instance, a prior study found no significant differences in outcome for therapy delivered for 48 versus 24 total hours [33]. In contrast, the findings from this study demonstrated significant incremental improvement over time for each additional day of practice, even at the lower end of the practice frequency range. For example, practicing for even 2 days versus 1 day a week confers a modest benefit in treatment outcome, which is useful information for clinicians seeking to set practical and attainable goals for patients.

Taken together, the results of this study provide critical information regarding the optimal dosage for a self-managed digital intervention. Currently, there are few empirical guidelines available to rehabilitation professionals to guide dose prescriptions for any speech-language–focused behavioral intervention, and none of these are specific to self-managed therapy modalities. This study adds to the limited body of existing literature on dose articulation and is the first to focus specifically on dose comparisons for self-managed digital therapy. We anticipate that the results will inform future recommendations of optimal dosage, which is critically needed as the field of speech-language pathology and stroke rehabilitation makes increasing use of digital therapy technologies.

### Limitations

This study is not without limitations. For instance, users were not randomly assigned to their dosage frequency group but were binned according to their usage pattern documented in the Constant Therapy system. It is possible that users with less severe impairments (determined by the baseline skill domain score) self-selected into different dosage groups. However, post hoc tests revealed differences in baseline severity between the ≥5 days per week practice group and other practice groups, and not among the 1, 2, 3, or 4 day per week practice groups. Therefore, a difference in baseline severity is not likely to explain the stepwise, incremental effects of dosage frequency found in this study. To further interrogate this question of whether users with less severe impairments are self-selectively getting more exposure to the treatment, we conducted a follow-up correlational analysis comparing the baseline domain score and the total number of practice hours (per skill domain). This analysis revealed no significant relationship between baseline severity and total amount of exposure (Multimedia Appendix 1), indicating that patients with less severe impairments at baseline did not have more exposure to the treatment. Despite the fact that this linear relationship was nonsignificant, all statistical models included both the baseline domain score and total number of hours as covariates to account for any potential effects of baseline severity or total therapy exposure, respectively, on performance gains over the treatment period.

A second limitation of this study was the lack of detailed person-level factors that could influence intervention outcomes. Although the Constant Therapy digital health platform allows for the collection of a large amount of real-world data across several English-speaking countries, it is currently impossible to collect detailed demographic and assessment information from all individuals. Thus, although we have included basic demographic covariates such as age, time since stroke, sex, and a proxy measure for baseline severity in our analysis models, the models would likely be improved with more detailed information about diagnosis, performance on standardized assessment metrics of language (eg, Western Aphasia Battery-Revised) or global function (eg, National Institutes of Health Stroke Scale and Modified Rankin Scale), concurrent medical and cognitive comorbidities, and psychosocial factors. A related limitation is the lack of information available in this data set regarding users’ access to direct therapy services. It is likely that for some users, the app-based regimen was used in conjunction with more traditional, in-person SLT, whereas for others, the app constituted the primary or singular mode of therapy. Systematic differences across the dosage groups in amounts of outside (ie, non–app-based) therapy received have the potential to influence observed results, as it is possible that frequent users of the app may also be receiving greater amounts of outside therapy, thus complicating the attribution of performance gain to a greater frequency of in-app practice.

Finally, although designed to be conservative estimates of therapeutic progress, we note that the skill domain scores used in this study are first-order approximations of functioning within a target skill domain. Improved approximations and validation against standardized assessments are the focus of ongoing and future work.

## Appendix

Multimedia Appendix 1 The linear mixed-effects models selection process, detailed statistical results, and supplemental analyses.

## Abbreviations

LMM: linear mixed-effects model
SLT: speech-language therapy
