# Supplementary material for: Dosage Frequency Effects on Treatment Outcomes Following Self-managed Digital Therapy: Retrospective Cohort Study
Source: J Med Internet Res. 2022 Jul 20;24(7):e36135. doi: 10.2196/36135 (PMC9350823; doi:10.2196/36135)
Supplement: Multimedia Appendix 1 [file jmir_v24i7e36135_app1.docx]

**Supplementary Table S1**. Linear mixed effects models building and selection process

| Model Specification | Model Name | Nested/ simpler model | Fixed Effects (FE) | Random Effects (RE) |  | | | LRT (v. nested) | | |
| --- | --- | --- | --- | --- | --- | --- | --- | --- | --- | --- |
|  |  |  |  |  | AIC | BIC | Marginal R2/ Conditional R2 | df | X2 | p |
| RE intercepts | RE0 | -- | -- | 1\|Patient  1\|Domain | -93230 | -93191 | 0.000 / 0.561 | -- | | |
| RE slopes/intercepts | **RE1** | RE0 | -- | 1 + Week\|Patient  1 + Week\|Domain | -100169 | -100092 | 0.000 / 0.591 | 4 | 6947.5 | <.001 |
| RE slopes/intercepts | RE2 | RE1 | -- | 1 + Dosage Group* Week\|Patient  1 + Dosage Group* Week\|Domain | Model did not converge | | | | | |
| FE main effects | M1 | RE1 | Week + Dosage Group + Total Hours + Baseline Domain Score + Age + Sex + Chronicity | 1 + Week\|Patient  1 + Week\|Domain | -151485 | -151302 | 0.443 / 0.680 | 11 | 51338 | <.001 |
| FE Two-way interactions | **M2** | M1 | Week*(Dosage Group + Total Hours) + Baseline Domain Score + Age + Sex + Chronicity | ‘’’’ | -151573 | -151342 | 0.446 / 0.681 | 5 | 97.943 | <.001 |
| N total observations = 111768; N patients = 2249; N domains = 13  Bold text indicates the optimal models selected for random effects structure (RE1) and overall final model incorporating random and fixed effects (M2) | | | | | | | | | | |

**Supplementary Table S2**. Standardized between-groups effect sizes for baseline domain score, total cumulative hours

|  |  | Standardized Effect Size  (LCL, UCL) | |
| --- | --- | --- | --- |
| Contrast |  | Baseline Domain Score | Total Hours |
| 1 day/week | 2 days/week | 0.0002 (-0.03, 0.03) | -0.07 (-0.1, -0.04) |
|  | 3 days/week | -0.006 (-0.04, 0.03) | -0.13 (-0.16, -0.1) |
|  | 4 days/week | -0.05 (-0.09, -0.02) | -0.17 (-0.21, -0.13) |
|  | 5 days/week | -0.09 (-0.13, -0.05) | -0.31 (-0.35, -0.27) |
| 2 days/week | 3 days/week | -0.006 (-0.03, 0.02) | -0.06 (-0.09, -0.04) |
|  | 4 days/week | -0.05 (-0.09, -0.02) | -0.10 (-0.13, -0.07) |
|  | 5 days/week | -0.09 (-0.13, -0.05) | -0.24 (-0.28, -0.2) |
| 3 days/week | 4 days/week | -0.05 (-0.08, -0.02) | -0.04 (-0.06, -0.008) |
|  | 5 days/week | -0.08 (-0.12, -0.05) | -0.18 (-0.21, -0.14) |
| 4 days/week | 5 days/week | -0.04 (-0.07, -0.002) | -0.14 (-0.17, -0.11) |
| LCL= Lower confidence limit; UCL=Upper confidence limit  Degrees-of-freedom method: inherited from asymptotic when re-gridding  Confidence level used: 0.95 | | | |

**Supplementary Table S3**. LMM results for individual subdomains (significant time×dosage group effect)

|  | *Arithmetic* | *Aud. Comp.* | *Aud. Memory* | *Naming* | *Quantitative* | *Reading* | *Visual Memory* | *Visuospatial* | *Writing* |
| --- | --- | --- | --- | --- | --- | --- | --- | --- | --- |
| Predictors | Est.  (SE) | Est.  (SE) | Est.  (SE) | Est.  (SE) | Est.  (SE) | Est.  (SE) | Est.  (SE) | Est.  (SE) | Est.  (SE) |
| (Intercept) | -1.17×10^-2^  (1.59×10^-2^) | 1.91×10^-3^  (9.14×10^-3^) | 2.85×10^-4^  (6.88×10^-3^) | **9.57×10^-2***^**  (1.78×10^-2^) | 2.12×10^-2^  (1.65×10^-2^) | 7.78×10^-3^  (1.34×10^-2^) | **4.89×10^-2***^**  (1.04×10^-2^) | **9.91×10^-2***^**  (1.24×10^-2^) | **6.88×10^-2**^**  (2.46×10^-2^) |
| Week | 3.70×10^-3^  (2.02×10^-3^) | **6.99×10^-3***^**  (1.09×10^-3^) | **7.47×10^-3***^**  (8.65×10^-4^) | -4.05×10^-3^  (2.64×10^-3^) | **8.33×10^-3***^**  (2.12×10^-3^) | **7.24×10^-3***^**  (1.51×10^-3^) | **8.94×10^-3***^**  (1.23×10^-3^) | **1.16×10^-2***^**  (1.29×10^-3^) | -2.92×10^-3^  (2.68×10^-3^) |
| Dosage Group [2] | -9.64×10^-3^ (7.48×10^-3^) | -6.81×10^-4^  (4.57×10^-3^) | 2.94×10^-3^  (3.54×10^-3^) | -3.08×10^-3^  (9.38×10^-3^) | 7.73×10^-3^  (8.42×10^-3^) | 6.53×10^-3^  (6.34×10^-3^) | **1.56×10^-2**^**  (5.23×10^-3^) | 8.17×10^-3^  (5.93×10^-3^) | -9.65×10^-3^  (1.17×10^-2^) |
| Dosage Group [3] | 1.65×10^-3^ (8.75×10^-3^) | 8.08×10^-3^  (4.88×10^-3^) | **8.86×10^-3*^**  (3.96×10^-3^) | -1.11×10^-2^  (9.87×10^-3^) | 1.14×10^-3^  (9.13×10^-3^) | 9.08×10^-3^  (6.81×10^-3^) | **1.58×10^-2**^**  (5.54×10^-3^) | **2.55×10^-2**^**  (6.53×10^-3^) | -1.63×10^-2^  (1.28×10^-2^) |
| Dosage Group [4] | 1.74×10^-2^ (9.68×10^-3^) | 4.79×10^-3^  (5.61×10^-3^) | 8.49×10^-3^  (4.41×10^-3^) | **-2.99×10^-2**^**  (1.07×10^-2^) | 1.17×10^-2^  (1.02×10^-2^) | 1.18×10^-2^  (7.61×10^-3^) | **2.05×10^-2**^**  (6.28×10^-3^) | **3.54×10^-2**^**  (7.44×10^-3^) | -2.63×10^-2^  (1.44×10^-2^) |
| Dosage Group [5+] | -9.58×10^-3^ (8.49×10^-3^) | **1.51×10^-2**^**  (5.35×10^-3^) | **1.72×10^-2***^**  (4.20×10^-3^) | **-2.99×10^-2**^**  (1.06×10^-2^) | -3.98×10^-3^  (9.90×10^-3^) | **1.59×10^-2*^**  (7.42×10^-3^) | **1.98×10^-2***^**  (5.63×10^-3^) | **4.08×10^-2***^**  (6.71×10^-3^) | -2.67×10^-2^  (1.41×10^-2^) |
| Total Hours | 2.10×10^-4^ (4.02×10^-4^) | 1.64×10^-4^  (1.46×10^-4^) | **5.12×10^-4***^**  (1.33×10^-4^) | 5.80×10^-5^  (4.82×10^-4^) | -2.10×10^-5^  (1.07×10^-4^) | -2.90×10^-5^  (8.90×10^-5^) | 1.41×10^-4^  (8.30×10^-5^) | -1.00×10^-6^  (4.40×10^-5^) | 1.28×10^-4^  (3.14×10^-4^) |
| Domain Score Baseline | **9.96×10^-1***^** (1.26×10^-2^) | **1.01^***^**  (1.22×10^-2^) | **1.05^***^**  (1.14×10^-2^) | **8.16×10^-1***^**  (1.25×10^-2^) | **9.21×10^-1***^**  (1.40×10^-2^) | **9.22×10^-1***^**  (1.39×10^-2^) | **9.12×10^-1***^**  (1.58×10^-2^) | **8.55×10^-1***^**  (1.38×10^-2^) | **8.87×10^-1***^**  (1.74×10^-2^) |
| Age | 1.03×10^-4^ (2.08×10^-4^) | -3.20×10^-5^  (1.19×10^-4^) | -8.70×10^-5^  (9.40×10^-5^) | -1.10×10^-4^  (2.25×10^-4^) | 6.60×10^-5^  (2.23×10^-4^) | 2.48×10^-4^  (1.65×10^-4^) | **-4.03×10^-4**^**  (1.30×10^-4^) | **-6.53×10^-4***^**  (1.51×10^-4^) | -5.70×10^-5^  (2.99×10^-4^) |
| Sex [Male] | 1.74×10^-2^ (9.68×10^-3^) | 2.00×10^-3^  (3.19×10^-3^) | 2.39×10^-3^  (2.52×10^-3^) | -8.52×10^-3^  (6.12×10^-3^) | 1.09×10^-2^  (6.01×10^-3^) | 3.41×10^-3^  (4.44×10^-3^) | 4.47×10^-3^  (3.51×10^-3^) | **8.91×10^-3*^**  (4.12×10^-3^) | -2.26×10^-3^  (8.33×10^-3^) |
| Sex [Not Spec.] | -1.06×10^-2^ (4.01×10^-2^) | -2.35×10^-2^  (3.22×10^-3^) | 1.26×10^-2^  (1.58×10^-2^) | 7.18×10^-3^  (6.64×10^-2^) | 8.14×10^-3^  (5.85×10^-2^) | 8.02×10^-3^  (4.08×10^-2^) | -1.65×10^-2^  (2.41×10^-2^) | 7.87×10^-3^  (2.31×10^-2^) | 3.99×10^-2^  (1.03×10^-1^) |
| Chronicity [Acute] | 6.69×10^-3^ (5.35×10^-3^) | 2.50×10^-5^  (3.19×10^-3^) | **5.80×10^-3*^**  (2.57×10^-3^) | 7.70×10^-3^  (6.11×10^-3^) | 2.98×10^-3^  (6.12×10^-3^) | **1.28×10^-2**^**  (4.41×10^-3^) | **1.06×10^-2**^**  (3.53×10^-3^) | **1.01×10^-2*^**  (4.12×10^-3^) | -6.76×10^-3^  (8.20×10^-3^) |
| Week* DG[2] | **5.93×10^-3*^** (2.55×10^-3^) | **3.58×10^-3*^**  (1.40×10^-3^) | **2.29×10^-3*^**  (1.11×10^-3^) | -1.72×10^-3^  (3.36×10^-3^) | 1.04×10^-3^  (2.75×10^-3^) | 2.53×10^-3^  (1.91×10^-3^) | **3.40×10^-3*^**  (1.53×10^-3^) | **3.27×10^-3*^**  (1.65×10^-3^) | 5.21×10^-3^  (3.37×10^-3^) |
| Week* DG[3] | **1.03×10^-2***^** (2.94×10^-3^) | **6.44×10^-3***^**  (1.47×10^-3^) | **4.04×10^-3**^**  (1.21×10^-3^) | 4.93×10^-3^  (3.51×10^-3^) | 5.17×10^-3^  (2.94×10^-3^) | 3.94×10^-3^  (2.03×10^-3^) | 2.48×10^-3^  (1.60×10^-3^) | 3.50×10^-3^  (1.78×10^-3^) | **1.08×10^-2**^**  (3.66×10^-3^) |
| Week* DG[4] | **1.39×10^-2***^** (3.26×10^-3^) | **6.85×10^-3***^**  (1.67×10^-3^) | **4.86×10^-3***^**  (1.34×10^-3^) | **9.22×10^-3*^**  (3.75×10^-3^) | **7.60×10^-3*^**  (3.30×10^-3^) | **8.17×10^-3***^**  (2.24×10^-3^) | **5.43×10^-3**^**  (1.81×10^-3^) | **4.16×10^-3*^**  (2.00×10^-3^) | **1.08×10^-2**^**  (4.09×10^-3^) |
| Week* DG[5+] | **1.29×10^-2***^** (2.85×10^-3^) | **7.91×10^-3***^**  (1.60×10^-3^) | **6.32×10^-3***^**  (1.28×10^-3^) | 6.04×10^-3^  (3.73×10^-3^) | **8.42×10^-3**^**  (3.17×10^-3^) | **9.25×10^-3***^**  (2.18×10^-3^) | **6.07×10^-3***^**  (1.61×10^-3^) | **5.66×10^-3**^**  (1.80×10^-3^) | 7.63×10^-3^  (3.97×10^-3^) |
| Week* Total Hours | -4.60×10^-5^ (1.31×10^-4^) | 7.40×10^-5^  (4.20×10^-5^) | **1.54×10^-4***^**  (3.90×10^-5^) | 3.00×10^-6^  (1.61×10^-43^) | -2.00×10^-5^  (3.30×10^-5^) | 5.00×10^-6^  (2.50×10^-5^) | 6.00×10^-6^  (2.30×10^-5^) | 1.10×10^-5^  (1.10×10^-5^) | 1.16×10^-4^  (8.80×10^-5^) |
| N_observations_ | 6407 | 13962 | 14393 | 8819 | 7115 | 9516 | 10574 | 11607 | 5755 |
| AIC | -13125 | -33779 | -39083 | -10318 | -11488 | -18714 | -25342 | -21143 | -8913 |
| Marginal/ Cond’l R2 | 0.65 / 0.93 | 0.56 / 0.91 | 0.62 / 0.90 | 0.49 / 0.85 | 0.57 / 0.89 | 0.53 / 0.87 | 0.52 / 0.87 | 0.53 / 0.83 | 0.53 / 0.91 |
| * *P* <.05; ** *P* <.01; *** *P* <.001 | | | | | | | | | |

**Supplementary Table S4**. LMM results for individual subdomains (non-significant time×dosage group effect)

| **Fixed Effects** | | | | |
| --- | --- | --- | --- | --- |
|  | *Analytical* | *Attention* | *Phonological Processing* | *Production* |
| Predictors | Est. (CI) | Est. (CI) | Est. (CI) | Est. (CI) |
| (Intercept) | **4.66×10^-2***^**  (1.07×10^-2^) | 2.55×10^-2^  (1.56×10^-2^) | **4.95×10^-2***^**  (1.78×10^-2^) | 3.54×10^-2^  (3.91×10^-2^) |
| Week | **8.95×10^-3***^**  (1.39×10^-3^) | **1.24×10^-2***^**  (1.63×10^-3^) | **6.87×10^-3*^**  (2.66×10^-3^) | **1.70×10^-2**^**  (6.33×10^-3^) |
| Dosage Group [2] | -2.68×10^-3^  (5.14×10^-3^) | 1.06×10^-2^  (6.94×10^-3^) | -1.22×10^-2^  (9.17×10^-3^) | 8.30×10^-3^  (2.13×10^-2^) |
| Dosage Group [3] | 8.94×10^-3^  (5.67×10^-3^) | **2.61×10^-2**^**  (8.14×10^-3^) | 1.54×10^-3^  (9.44×10^-3^) | 8.41×10^-3^  (2.30×10^-2^) |
| Dosage Group [4] | 8.80×10^-3^  (6.38×10^-3^) | **3.09×10^-3**^**  (6.38×10^-3^) | -5.03×10^-3^  (1.04×10^-2^) | 1.92×10^-3^  (2.53×10^-2^) |
| Dosage Group [5+] | 6.25×10^-3^  (5.84×10^-3^) | **2.77×10^-2**^**  (9.65×10^-3^) | -1.90×10^-2^  (1.01×10^-2^) | -1.88×10^-2^  (2.97×10^-2^) |
| Total Hours | 1.20×10^-5^  (6.40×10^-5^) | 8.70×10^-5^  (2.05×10^-4^) | 5.16×10^-4^  (6.72×10^-4^) | 2.14×10^-3^  (3.15×10^-3^) |
| Domain Score Baseline | **9.33×10^-1***^**  (1.28×10^-2^) | **9.60×10^-1***^**  (1.72×10^-2^) | **8.62×10^-1***^**  (1.81×10^-2^) | **8.18×10^-1***^**  (3.15×10^-2^) |
| Age | **-3.12×10^-4*^**  (1.35×10^-4^) | 2.72×10^-4^  (1.98×10^-4^) | -1.50×10^-5^  (2.33×10^-4^) | 8.19×10^-4^  (5.50×10^-4^) |
| Sex [Male] | 7.41×10^-4^  (3.70×10^-3^) | 8.17×10^-3^  (5.37×10^-3^) | 4.16×10^-3^  (6.19×10^-3^) | -1.49×10^-3^  (1.51×10^-2^) |
| Sex [Not Spec.] | -1.03×10^-2^  (2.40×10^-2^) | 5.03×10^-2^  (2.80×10^-2^) | -7.01×10^-2^  (7.40×10^-2^) | -- |
| Chronicity [Chronic] | 1.45×10^-3^  (3.69×10^-3^) | 6.60×10^-4^  (5.35×10^-3^) | **1.91×10^-2**^**  (6.19×10^-3^) | 2.90×10^-3^  (1.47×10^-2^) |
| Week* DG[2] | -5.99×10^-4^  (1.85×10^-3^) | 3.40×10^-3^  (2.17×10^-3^) | 3.25×10^-3^  (3.51×10^-3^) | -7.50×10^-5^  (8.16×10^-3^) |
| Week* DG[3] | -3.58×10^-4^  (2.01×10^-3^) | 1.06×10^-3^  (2.53×10^-3^) | -1.22×10^-3^  (3.59×10^-3^) | 1.09×10^-2^  (8.87×10^-3^) |
| Week* DG[4] | 3.15×10^-4^  (2.26×10^-3^) | -1.48×10^-3^  (2.98×10^-3^) | 4.24×10^-3^  (3.93×10^-3^) | -7.97×10^-4^  (9.44×10^-3^) |
| Week* DG[5+] | -2.02×10^-3^  (2.07×10^-3^) | 1.71×10^-3^  (2.99×10^-3^) | 7.13×10^-4^  (3.88×10^-3^) | 1.83×10^-2^  (1.13×10^-2^) |
| Week* Total Hours | -1.40×10^-5^ (2.20×10^-5^) | -7.10×10^-5^  (6.00×10^-5^) | -2.54×10^-4^  (2.54×10^-4^) | -1.44×10^-3^  (1.18×10^-3^) |
| **Model Fit** | | | | |
| N_observations_ | 9221 | 7429 | 5428 | 1542 |
| AIC | -21141 | -13768 | -9786 | -1759 |
| Marginal/ Cond’l R2 | 0.54 / 0.89 | 0.54 / 0.88 | 0.42 / 0.88 | 0.46 / 0.86 |
| * *P*<.05; ** *P* <.01; *** *P* <.001 | | | | |

**Supplementary Figure S1**. Amount of therapy exposure as a function of baseline severity


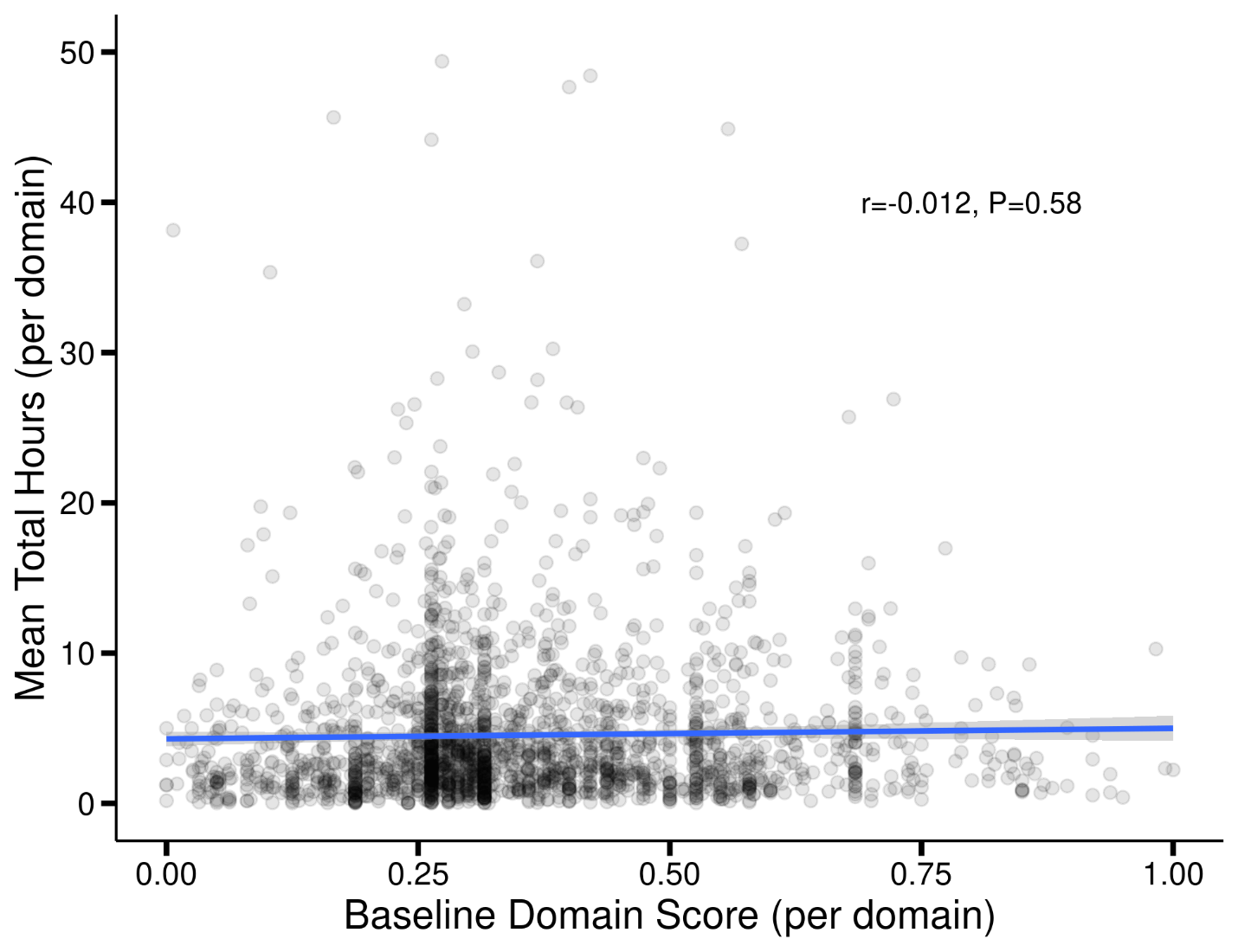


A Pearson correlation analysis revealed no significant relationship between the amount of therapy exposure (mean total hours, per domain) and individual users’ starting severity (baseline domain score, per domain). For better visualization, an upper y-axis limit = 50 is applied to this figure, leading to the exclusion of 33 individual datapoints. Though not displayed, these datapoints were included in the correlation analysis.

**Supplementary Figure S2**. Weekly change in domain score as a function of dosage frequency group, Naming and Writing domains


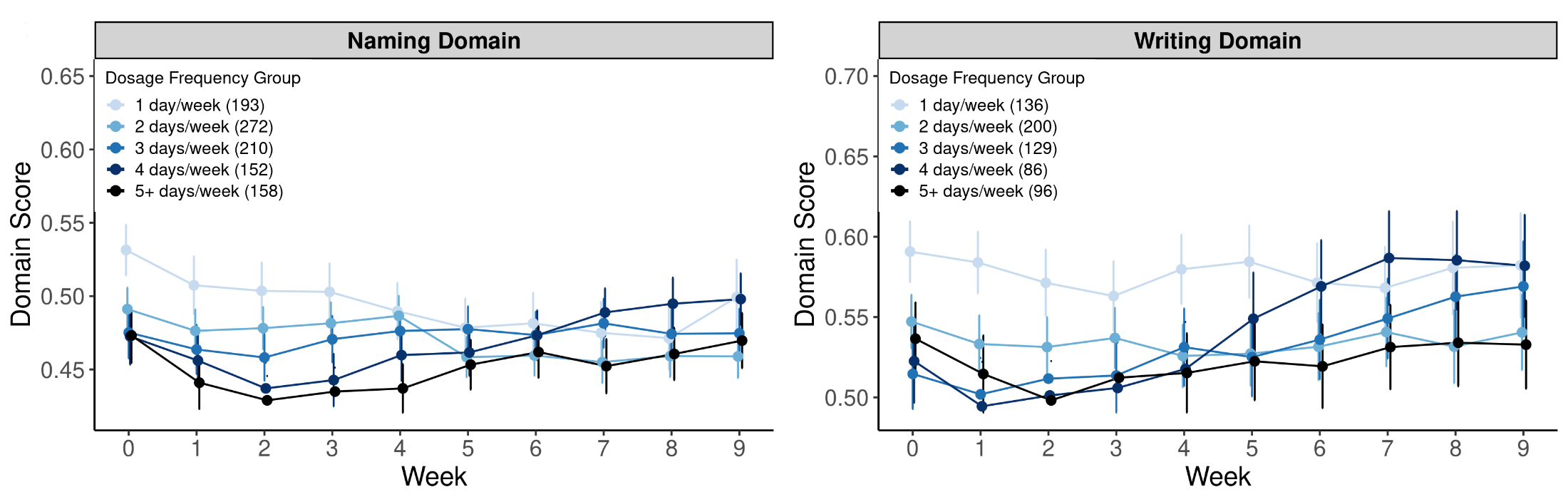


For the naming and writing domains, results showed a significant time×dosage frequency group interaction, though post-hoc comparisons revealed significantly greater rates of change in domain score only for practice frequencies of 3 and/or 4 days/week as compared to one day per week.
